# Heme Oxygenase-1 Regulates the Progression of K/BxN Serum Transfer Arthritis

**DOI:** 10.1371/journal.pone.0052435

**Published:** 2012-12-20

**Authors:** Rita Brines, Nuria Maicas, María Luisa Ferrándiz, Agnieszka Loboda, Alicja Jozkowicz, Jozef Dulak, María José Alcaraz

**Affiliations:** 1 Department of Pharmacology and IDM, University of Valencia, Burjasot, Valencia, Spain; 2 Department of Medical Biotechnology, Faculty of Biochemistry, Biophysics and Biotechnology, Jagiellonian University, Krakow, Poland; Chang Gung University, Taiwan

## Abstract

**Background:**

Heme oxygenase-1 (HO-1) is induced in many cell types as a defense mechanism against stress. We have investigated the possible role of endogenous HO-1 in the effector phase of arthritis using the K/BxN serum transfer model of arthritis in HO-1 heterozygous and homozygous knock-out mice.

**Methodology/Principal Findings:**

Arthritis was induced in C57/Black-6 xFVB (HO-1^+/+^, HO-1^+/−^ and HO-1^−/−^) mice by intraperitoneal injection of 150 µl serum from arthritic K/BxN mice at days 0 and 2. Blood was collected and animals were sacrificed at day 10. Histological analysis was performed in ankle sections. The levels of inflammatory mediators were measured in serum and paw homogenates by enzyme-linked immunosorbent assay or Multiplex technology. The incidence of arthritis was higher in HO-1^+/−^ and HO-1^−/−^ groups compared with HO-1^+/+^. The inflammatory response was aggravated in HO-1^+/−^ mice as shown by arthritic score and the migration of inflammatory cells that could be related to the enhancement of CXCL-1 production. In addition, the HO-1^+/−^ group showed proteoglycan depletion significantly higher than HO-1^+/+^ mice. Serum levels of matrix metalloproteinase-3, monocyte chemotactic protein-1, plasminogen activator inhibitor-1, E-selectin and intercellular adhesion molecule-1 were increased in arthritic HO-1^−/−^ mice, whereas vascular endothelial growth factor and some cytokines such as interferon-γ showed a reduction compared to HO-1^+/+^ or HO-1^+/−^ mice. In addition, down-regulated gene expression of ferritin, glutathione S-reductase A1 and superoxide dismutase-2 was observed in the livers of arthritic HO-1^+/−^ animals.

**Conclusion/Significance:**

Endogenous HO-1 regulates the production of systemic and local inflammatory mediators and plays a protective role in K/BxN serum transfer arthritis.

## Introduction

Results of previous studies indicate that heme oxygenase-1 (HO-1) induction plays a role in protecting tissues against injury through the modulation of oxidative stresss or cellular activation [1]. Conversely, HO-1 deficiency has revealed an important role for this enzyme in immune responses [2]. Expression levels of HO-1 are excessively increased in patients with some inflammatory diseases suggesting its participation in the development of systemic inflammation [3]. However, little is known about the role of HO-1 in arthritic disorders. An association of *HO-1* promoter polymorphism with rheumatoid arthritis (RA) has been reported [4] and patients with the SS genotype, resulting in a higher HO-1 expression, may have a better long-term radiographic outcome despite disease activity [5]. HO-1 protein is expressed in synovial tissues of RA patients [6] and its presence in synovial fluid may be a marker of joint inflammation [7] Nevertheless, treatment of RA patients with tumor necrosis factor α (TNF-α) antagonists may block the TNF-α-dependent suppression of HO-1 expression in peripheral blood cells, resulting in amelioration of inflammation [8].

There is emerging evidence that pharmacological induction of HO-1 results in anti-inflammatory effects in models of RA [9], [10]. Metabolites derived from HO-1 activity such as biliverdin/bilirubin or carbon monoxide (CO) may be responsible for the anti-inflammatory actions of this enzyme. In particular, CO may mediate the main actions of HO [11], [12]. In arthritic processes, we have shown that administration of the CO-releasing molecule CORM-3 results in the down-regulation of the inflammatory response in the collagen-induced [13] and K/BxN serum transfer [14] arthritis models.

Metalloporphyrin inhibitors of HO-1 exhibit a number of nonspecific effects [15], [16] and therefore they have limitations in assessing the role of endogenous HO-1. However, the use of genetically deficient mice can provide a better insight into the possible regulatory effects of HO-1 in inflammatory responses. Studies in HO-1^−/−^ mice have revealed that HO-1 deficiency favors the production of Th1 cytokines by stimulated splenocytes suggesting a pro-inflammatory tendency [17]. In addition, the apparent defect in immune regulation in HO-1^−/−^ mice may be related to the inhibition of Treg suppressive activity [18]. Transfer of serum from K/BxN transgenic mice into healthy animals induces an autoimmune and inflammatory response mediated by IgG1 autoantibodies with a number of similarities to human RA [19], [20]. In this study, we have investigated whether endogenous HO-1 would play a role in the effector phase of inflammatory arthritis by inducing K/BxN serum transfer arthritis in HO-1 wild type (HO-1^+/+^), heterozygous (HO-1^+/−^) and homozygous knock out (HO-1^−/−^) mice.

## Results

### Evolution of Arthritis

**Figure 1:**
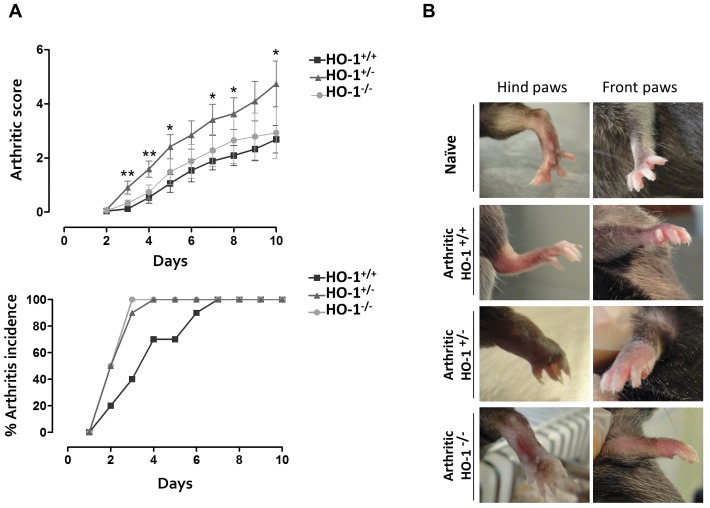
Evolution of arthritis. A, Time-course of clinical score values (mean ± SEM) and arthritis incidence (% of mice with arthritis), (*n* = 10 animals per group). *P<0.05, **P<0.01, ***P<0.001 with respect to the arthritic HO-1^+/+^ group. **B**, Pictures of representative paws taken at the end of the experiment (day 10).

Figure 1A shows the time-course of macroscopic score values and arthritis incidence. Representative images of hind paws and front paws from all experimental groups at the end of the experiment are shown in Figure 1B. In mice injected with K/BxN serum inflammation increased progressively with time. We noted that HO-1^+/−^ and HO-1^−/−^ mice developed accelerated arthritis compared with HO-1^+/+^ animals. Arthritic score values were also increased in both groups although they reached statistical significance in HO-1^+/−^ mice only.

### Histological Analysis

**Figure 2:**
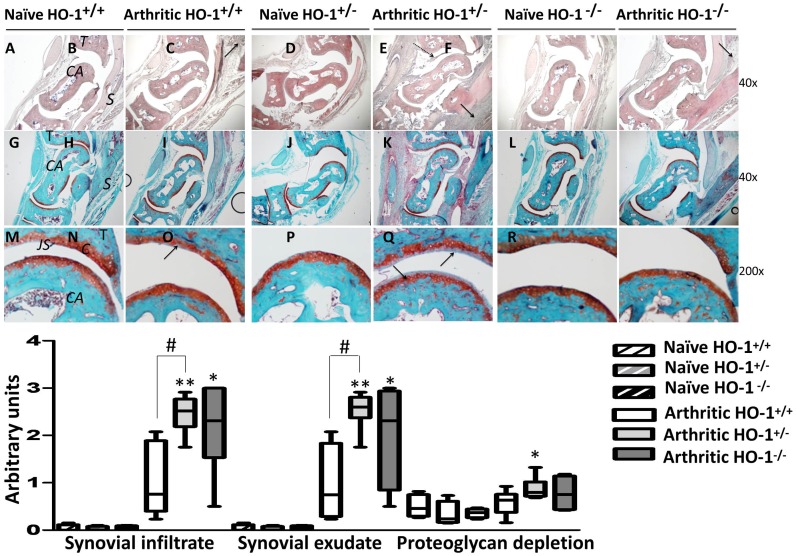
Histological analysis of frontal sections of ankle joints at day 10. **A–F:** hematoxylin and eosin-stained sections, and **G–R**: safranin O and fast green-stained sections. *C = cartilage; CA = calcaneous; JS = joint space; S = synovium; T = tibia.* **B, D** and **F**: solid arrows indicate areas of cell infiltration and broken arrows indicate exudate. **N** and **P**: solid arrows indicate proteoglycan depletion in cartilage matrix. The histological scores are presented as mean ± SEM (*n* = 4–10). *P<0.05, **P<0.01, each arthritic group compared to its respective naïve group. #*P*<0.05 with respect to arthritic HO-1^+/+^ group.

In arthritic animals, ankle sections showed the presence of inflammatory cells, synovial hyperplasia and cartilage destruction (Figure 2). There was a two-fold increase in synovial infiltration and accumulation of inflammatory cells in joint spaces in arthritic HO-1^+/−^ compared to HO-1^+/+^ mice. The values for HO-1^−/−^ animals also increased although to a lower extent. In addition, the HO-1^+/−^ group showed proteoglycan depletion scores higher than those of HO-1^+/+^ mice.

### Blood Determinations

**Figure 3:**
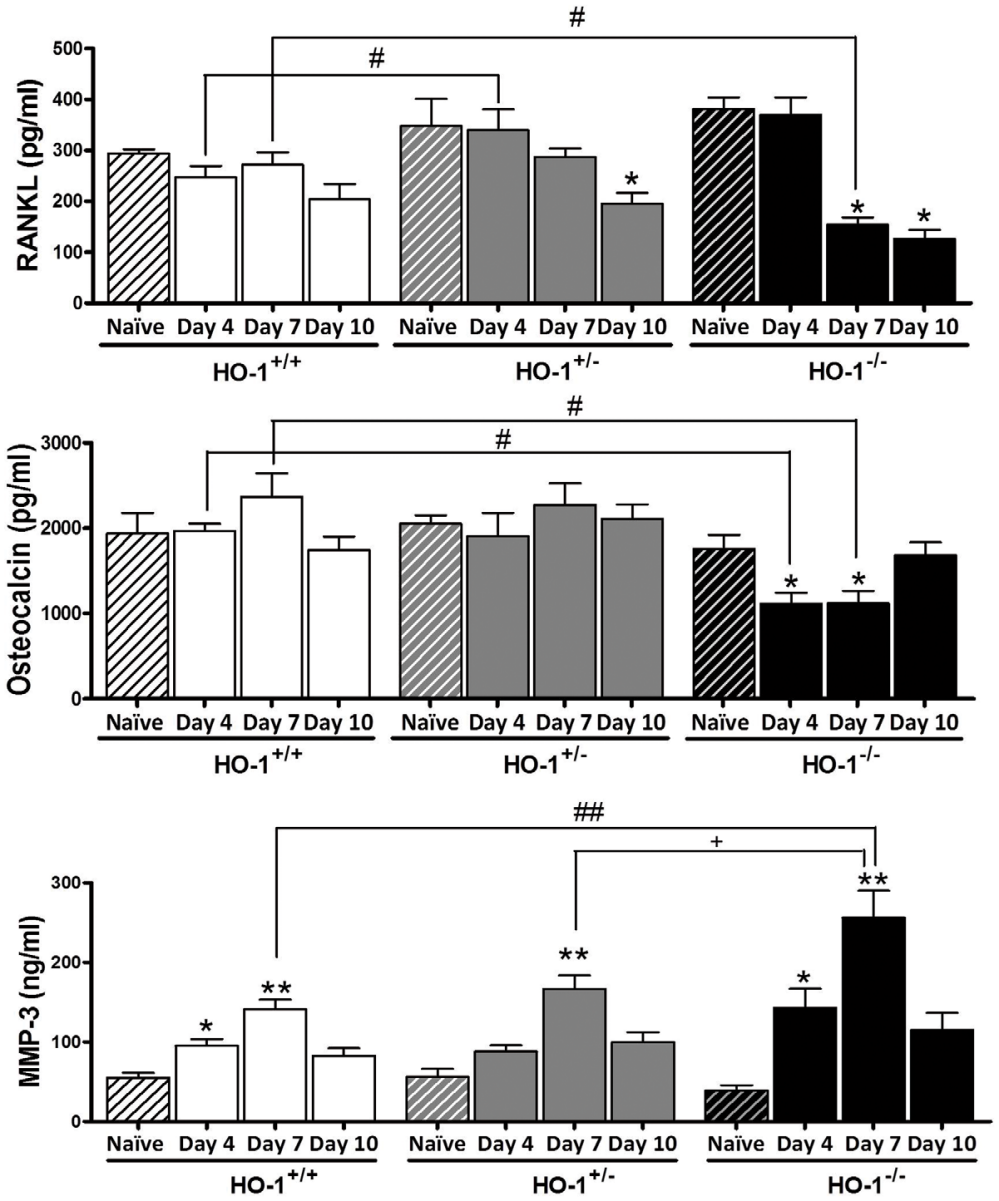
Time-course of RANKL, osteocalcin and MMP-3 levels in serum. Data represent mean ± SEM, *n* = 4–10. ^*^ *P*<0.05, ^**^ *P*<0.01 each arthritic group compared with its respective naïve group, ^#^ *P*<0.05,^ ##^ *P*<0.01 with respect to arthritic HO-1^+/+^ group.

**Figure 4:**
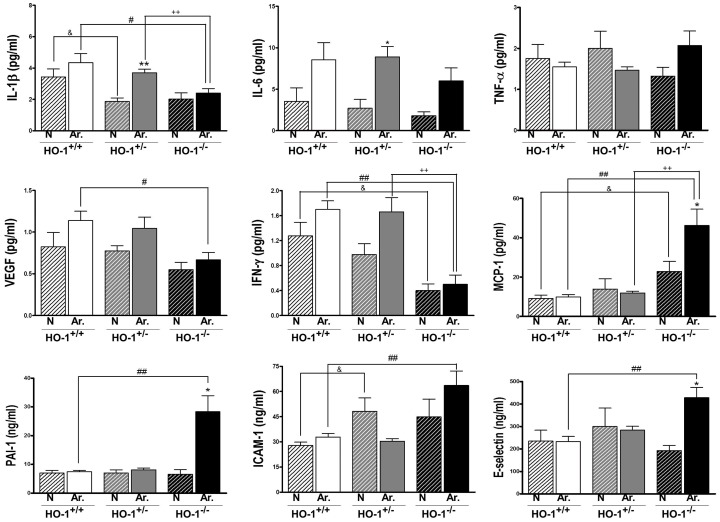
Levels of inflammatory mediators in serum at day 10. Data represent mean ± SEM, *n* = 4–10. N (naïve); Ar. (arthritic). ^*^ *P*<0.05, ^**^ *P*<0.01 each arthritic group compared with its respective naïve group, ^#^ *P*<0.05,^ ##^ *P*<0.01 with respect to arthritic HO-1^+/+^ group, ^++^ *P*<0.01 with respect to arthritic HO-1^+/−^ group, ^&^ *P*<0.05 with respect to naïve HO-1^+/+^ group.

Naïve HO-1^−/−^ mice showed increased counts of lymphocytes, granulocytes and monocytes with respect to HO-1^+/+^ or HO-1^+/−^ animals (Table 1) which were also observed after arthritis induction. In contrast, a small reduction in leukocyte numbers was observed in arthritic mice with one HO-1 allele with respect to arthritic HO-1^+/+^ controls. In addition, arthritis resulted in the production of anemia accompanied by increased platelet counts in all groups although these effects were more pronounced in HO-1^−/−^ animals. Other blood parameteres were not significantly different in either mice lacking both or one HO-1 allele with respect to HO-1^+/+^ animals (data not shown). Naïve HO-1^+/−^ and HO-1^−/−^ mice showed a tendency to increased serum levels of receptor activator for nuclear factor κB ligand (RANKL) in comparison with HO-1^+/+^ (Figure 3). Induction of arthritis led to a time-dependent reduction in RANKL which was significant for HO-1^+/−^ mice at day 10 and HO-1^−/−^ animals at days 7 and 10. We also observed that osteocalcin levels were significantly reduced at days 4 and 7 in HO-1^−/−^ mice with respect to HO-1^+/+^. Matrix metalloproteinase-3 (MMP-3) was significantly increased in the last group at days 4 and 7 after serum transfer in comparison with naïve mice. In HO-1 deficient mice, MMP-3 showed a similar profile with time and its levels were significantly higher in HO-1^−/−^ mice than in HO-1^+/+^ or HO-1^+/−^ animals at day 7. As shown in Figure 4, the levels of a number of inflammatory mediators measured in serum at the end of the experiment (day 10) were modified in HO-1 deficient animals. The levels of interleukin(IL)-1β, interferon-γ (IFN-γ) and vascular endothelial growth factor (VEGF) after arthritis induction were lower in HO-1^−/−^ animals with respect to HO-1^+/+^ or HO-1^+/−^ mice. IL-17 was not significantly modified at this time point (data not shown) but its levels measured at day 7 showed a similar profile with values (mean±SEM) for arthritic animals of 4.8±0.7 pg/ml (HO-1^+/+^), 3.4±0.5 pg/ml (HO-1^+/−^) and 2.5±0.6 pg/ml (HO-1^−/−^, *P*<0.05 with respect to HO-1^+/+^). In contrast, arthritic HO-1 deficient mice showed higher levels of monocyte chemotactic protein-1 (MCP-1), intercellular adhesion molecule-1 (ICAM-1), E-selectin and plasminogen activator inhibitor-1 (PAI-1) with respect to arthritic HO-1^+/+^ or HO-1^+/−^ animals. Serum ICAM-1 was also enhanced in naïve HO-1^+/−^ and HO-1^−/−^ mice compared to naïve HO-1^+/+^ animals. IFN-γ levels in naïve HO-1^−/−^ mice were lower than those of naïve HO-1^+/+^ whereas MCP-1 levels were significantly augmented. In addition, naïve mice lacking one or two HO-1 alleles showed IL-1β levels lower than HO-1^+/+^ animals and arthritis induction led to a significant increase of this cytokine in HO-1^+/−^ animals.

**Table 1:** Blood cell analysis.

			NAÏVE MICE				
	WBC(10^3^/µl)	LYMPHOCYTES(10^3^/µl)	MONOCYTES(10^3^/µl)	GRANULOCYTES(10^3^/µl)	RBC(10^6^/µl)	HGB(g/dl)	PLT(10^5^/µl)
HO-1^+/+^	17.1±4.2	13.5±3.9	0.7±0.2	3.9±0.4	7.2±0.8	17.3±3.3	10.5±0.7
HO-1^+/−^	17.1±2.2	13.5±3.0	0.6±0.1	3.0±0.6	6.9±1.0	18.0±2.0	9.2±3.2
HO-1^−/−^	25.7±3.4#++	19.1±2.2##+	19.1±2.2##+	5.4±1.3#+	7.2±1.0	17.0±2.0	9.6±2.7
			**ARTHRITIC MICE**				
	**WBC** **(10^3^/µl)**	**LYMPHOCYTES** **(10^3^/µl)**	**MONOCYTES** **(10^3^/µl)**	**GRANULOCYTES** **(10^3^/µl)**	**RBC** **(10^6^/µl)**	**HGB** **(g/dl)**	**PLT** **(10^5^/µl)**
HO-1^+/+^	16.9±3.5	12.3±2.7	0.7±0.2	3.9±1.0	5.2±0.7**	14.8±1.7	12.6±2.3
HO-1^+/−^	12.9±3.2*#	9.4±2.0**#	0.5±0.2	2.9±1.2	5.5±0.6*	14.7±1.4**	14.0±1.1**
HO-1^−/−^	33.3±14.3#+	22.1±8.6#++	1.5.1±0.8##++	9.7±5.7	5.5±0.8**	12.3±1.8**#+	15.5±1.5**#

Results show mean±S.D. *P<0.05, **P<0.01 *versus* the corresponding naïve group; ^#^ *P*<0.05, ^##^ *P*<0.01 *versus* HO-1^+/+^ in the same group; +*P*<0.05, ++*P*<0.01 *versus* HO-1^+/−^ in the same group.

### Levels of Mediators in Ankles

**Figure 5:**
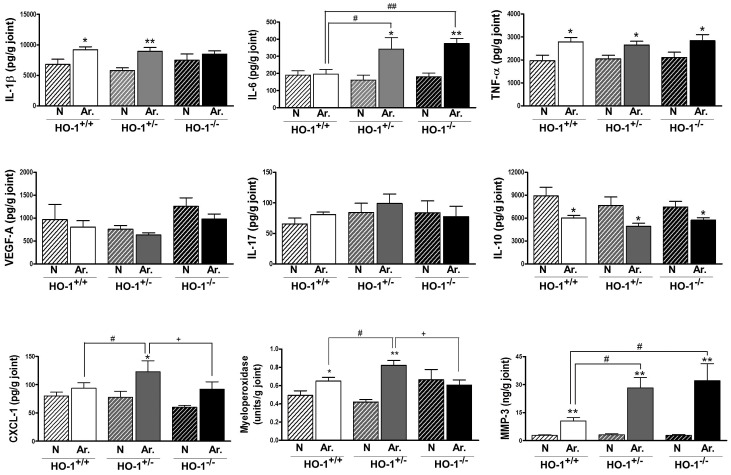
Levels of inflammatory mediators in ankle homogenates at day 10. Data represent mean ± SEM, *n* = 4–10. N (naïve); Ar. (arthritic). ^*^ *P*<0.05, ^**^ *P*<0.01 each arthritic group compared with its respective naïve group, ^#^ *P*<0.05,^ ##^ *P*<0.01 with respect to arthritic HO-1^+/+^ group, ^+^ *P*<0.05 with respect to arthritic HO-1^+/−^ group.

The production of cytokines and chemokines in the joint plays an important role in inflammatory arthritis. Local production of inflammatory mediators was examined in ankle homogenates. Induction of arthritis in wild type mice resulted in increased levels of IL-1β, TNF-α and MMP-3 at day 10 (Figure 5). There were no differences in IL-1β, TNF-α, IL-17 or VEGF-A levels between the different genotypes. In arthritic animals, IL-6 and MMP-3 significantly increased in HO-1^+/−^ and HO-1^−/−^ mice with respect to HO-1^+/+^ group. In contrast, induction of arthritis decreased IL-10 in all three genotypes. In addition, we observed in arthritic animals that CXCL-1 levels were significantly enhanced in HO-1^+/−^ mice with respect to wild type or HO-1^−/−^ mice. We also determined the levels of myeloperoxidase activity as a marker of neutrophils. No significant differences were observed between naïve HO-1^+/+^ and HO-1^+/−^ mice whereas naïve HO-1^−/−^ mice showed a tendency to increased values. The induction of arthritis resulted in a significant enhancement in myeloperoxidase activity with respect to the corresponding naïve animals, in HO-1^+/+^ and HO-1^+/−^ mice but not in HO-1^−/−^ mice. Myeloperoxidase activity was significantly higher in arthritic HO-1^+/−^ mice compared with arthritic HO-1^+/+^ or HO-1^−/−^ groups.

### Expression of Antioxidant Genes

**Figure 6:**
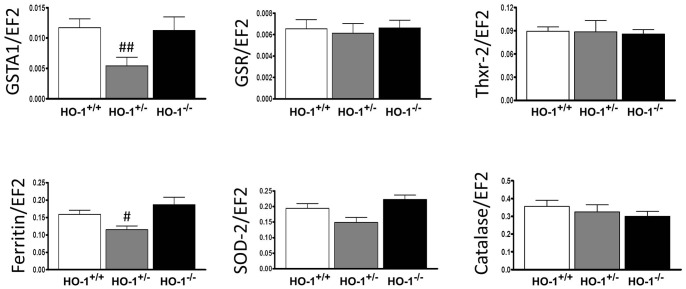
Gene expression levels at day 10. Relative mRNA levels of glutathione S-transferase A1 (GSTA1), glutathione reductase (GSR), ferritin, thioredoxin-2 (Thxr-2), superoxide dismutase-2 (SOD-2) and catalase were measured in livers of arthritic mice at day 10 by real-time PCR. Results were normalized with respect to elongation factor 2 (EF2) and expressed as 2^−ΔCt^. Data represent mean ± SEM, *n* = 8–9. ^#^ *P*<0.05, ^##^ *P*<0.01 compared with arthritic wild type animals.

Because both partial and complete HO-1 deficiency led to increased susceptibility to arthritis but some effects were even more pronounced in HO-1^+/−^ animals we checked the expression of other than HO-1 antioxidant genes in arthritic animals of all genotypes. Using real-time PCR we analyzed the expression of thioredoxin-2 (Thrx-2), glutathione S*-*transferase-1 (GSTA-1), glutathione reductase (GR), superoxide dismutase-2 (SOD-2), ferritin and catalase in the livers of arthritic animals. Interestingly, we observed that in mice which lack one HO-1 allele, but not in the HO-1^−/−^ animals, the expression of some antioxidant genes was diminished. The decrease in ferritin and GSTA1 expression was statistically significant and there was a tendency for the down-regulation of SOD-2 (*P* = 0.07). On the other hand, the expression of Thrx-2, GSR and catalase was similar in mice of all genotypes (Figure 6). The observed changes in the HO-1^+/−^ animals after arthritis induction may suggest that increased susceptibility to arthritis development may result from the decreased systemic anti-oxidative response, not only HO-1 activity.

## Discussion

We have studied the consequences of HO-1 deficiency in an autoantibody-mediated model of RA that bypasses the immunologic phase of arthritis. Our results indicate that HO-1 deficiency aggravates the progression of K/BxN serum transfer arthritis. An earlier initiation of arthritis and higher arthritic score values were observed in HO-1^+/−^ and to a lower extent in HO-1^−/−^ mice compared to arthritic HO-1^+/+^ animals. This animal model is dependent on the presence of neutrophils and macrophages [20], [21] with an essential role of neutrophils in the establishment of immune complex-mediated arthritis [22]. The histopathological analysis of the present study confirmed the clinical findings and the higher inflammatory response in the joints of HO-1^+/−^ mice compared with HO-1^−/−^ and HO-1^+/+^ mice which would be dependent on the enhanced migration of neutrophils. These local effects could be related to the production of chemokines such as CXCL-1. It is interesting to note that this chemokine has been reported to mediate neutrophil recruitment to the joints of K/BxN serum transfer arthritis and its up-regulation in ankle and synovial fluid parallels the progression of disease [23]. Deficiency in HO-1 did not significantly affect basal expression of mediators in the joint. However, after induction of arthritis, we observed the local up-regulation of pro-inflammatory IL-6 and MMP-3 in both HO-1^+/−^ and HO-1^−/−^ animals with respect to wild type mice. HO-1 deficiency also affected serum levels of RANKL and osteocalcin in arthritic animals with stronger effects for the HO-1^−/−^ genotype. Our results thus suggest a role for HO-1 in osteoblast function during the course of arthritis.

Serum levels of IL-1β and IFN-γ were lower in arthritic HO-1^−/−^ animals compared with arthritic HO-1^+/−^ and HO-1^+/+^ mice. Given the important role of IL-1β in K/BxN inflammation [24], the absence of enhancing effects on this cytokine may contribute to the lower arthritic response observed in HO-1^−/−^ animals compared with HO-1^+/−^ mice. In addition, IFN-γ may play a complex role in this experimental model as administration of low doses of this cytokine exerts inhibitory effects on arthritis [25]. The absence of HO-1 resulted in higher serum MCP-1 levels compared to HO-1^+/+^ mice. This observation is consistent with previous studies showing that the protective effects of HO-1 in injured renal tissues may depend on HO-1 ability to restrain up-regulation of MCP-1 [26]. Induction of arthritis in HO-1^−/−^ mice but not in HO-1^+/−^ animals significantly increased serum levels of MMP-3, PAI-1, E-selectin and ICAM-1 whereas VEGF levels were lower than those of HO-1^+/+^ or HO-1^+/−^ mice. Similar observations on PAI-1 increased expression were made in mouse embryonic HO-1 deficient cells [27]. In HO-1^−/−^ mice, enhanced serum ICAM-1, E-selectin, and MMP-3 levels indicate the presence of vasculitis and systemic inflammation. Our data in this animal model of RA are in line with the results of previous studies showing that HO-1 exhibits anti-inflammatory and protective effects against organ injury by inhibiting the expression of adhesion molecules [28], [29] and MCP-1 [26] besides promoting vascular repair through VEGF production [30].

The mechanism by which HO-1 deficiency modulates the effector phase of arthritis is complex. In this study, HO-1^+/−^ and HO-1^−/−^ mice showed a higher susceptibility to develop arthritis after K/BxN serum transfer when compared with their wild type counterparts. Nevertheless, the inflammatory response in the joint was more aggressive in animals lacking one HO-1 allele with respect to complete HO-1 absence. To elucidate this effect we measured the expression of other than HO-1 antioxidant genes in the livers of the affected animals. Interestingly, we observed a significant reduction of ferritin and GSTA1 and a trend to down-regulation of SOD-2 expression in HO-1^+/−^ animals only. These genes are part of interactive defense mechanisms that allow adaptation to oxidative stress exposure and result in anti-inflammatory effects [31]–[34]. Such differential expression of antioxidant enzymes after stressful conditions in mice with different HO-1 levels was previously observed by us in another model [35]. Thus, in mice subjected to chemical induction of squamous cell carcinoma, the expression of catalase and GR was decreased only in HO-1^+/−^ mice and was not affected in HO-1 knock-out animals [35]. The partial loss of antioxidant and antiinflammatory protection in heterozygous HO-1 mice may contribute to the development of an inflammatory response more aggressive than in wild type mice. Nevertheless, the strong loss of HO-1 in knock-out mice may be a stimulus leading to the partial activation of other defense systems during stressful situations such as arthritis induction. As a result, the inflammatory response was up-regulated to a lower extent. These observations suggest that HO-1 knock-out mice might be able to develop compensatory mechanisms against stress, but the lack of only one allele of HO-1 may not be sufficient to initiate such a response.

Genetically decreased basal HO-1 levels in HO-1^+/−^ mice can represent a more physiological situation than total HO-1 deletion as HO-1 has a role in maintaining the body homeostasis. It is known that HO-1^−/−^ mice develop with time a number of changes indicating defects in immune regulation, the presence of a chronic inflammatory state and organ damage [17], [18], [36]. These results in the K/BxN serum transfer model of arthritis indicate that HO-1 absence deregulates homeostasis of leukocytes either in naïve or arthritic animals as well as vascular homeostasis leading to systemic inflammation during the development of the effector phase of arthritis which may influence the response to K/BxN serum. Therefore, an altered production of cytokines and chemokines which critically determine leukocyte circulating numbers, cell activation and tissue infiltration could promote systemic inflammatory changes in HO-1^−/−^ mice that may limit the joint response. In particular, the presence in these mice of high serum levels of MCP-1 and PAI-1 may determine vascular disfunction with recruitment of inflammatory cells into neointima, hyperplasia and a procoagulant state [26].

In conclusion, deficiency of HO-1 in heterozygous animals potentiates arthritis due to the up-regulation of cytokines and chemokines relevant for neutrophil migration and joint inflammation, whereas deletion of HO-1 results in systemic inflammation leading to a lower ability to potentiate local joint inflammation. Our findings point to a modulatory role of endogenous HO-1 in the effector phase of arthritis and provide new insights into the anti-inflammatory properties of HO-1.

## Methods

### Ethics Statement

All studies were performed in accordance with European Union regulations for the handling and use of laboratory animals. The protocols were approved by the institutional Animal Care and Use Committees (Jagiellonian University, Krakow, Poland and *Comité Etico de Bienestar y Experimentación Animal*, University of Valencia, Spain, A1264890323968).

### Induction of Arthritis

Experiments were performed on 20–27-week-old male C57/Black-6 xFVB (HO-1^+/+^, HO-1^+/−^ and HO-1^−/−^) mice generated from the HO-1^+/−^ breeding pair kindly gifted by Dr. Anupam Agarwal (Birmingham, AL, USA). Mice were genotyped three weeks after birth by PCR using tail DNA as previously described [36]. All mice were maintained in cages with a 12-hour light/dark cycle, at 22°C and free access to sterilized food and water. Mice were housed and cared for by the veterinary staff and were routinely screened for health status. Animals were assigned to experimental groups (*n* = 10 in arthritic groups and *n* = 4 in naïve groups): arthritic HO-1^+/+^, arthritic HO-1^+/−^, arthritic HO-1^−/−^, naïve HO-1^+/+^, naïve HO-1^+/−^ and naïve HO-1^−/−^. Arthritis was induced by intraperitoneal injection of serum (150 µl) from arthritic K/BxN mice (generated by crossing KRN-TCR-transgenic mice (B10.BR genetic background) with NOD mice) at days 0 and 2. Blood was collected from the retroorbital venous plexus at days 4, 7 and 10, as well as from tail at day 10, and animals were sacrificed by cervical dislocation at day 10. Ankles from randomly selected animals in each group were isolated for histological analysis and the rest of them were used for measurement of inflammatory markers.

### Arthritis Score

The clinical severity of arthritis was graded using a scale of 0–2 for each paw where 0 = uninflamed, 1 = mild, 1.5 = marked and 2 = severe inflammation, and the four-limb scores were summed to yield the clinical index. Scoring was performed by two independent observers without knowledge of the experimental groups.

### Histological Analysis

For standard histological assessment, isolated ankles were kept in 4% formalin in phosphate-buffered saline for 4 days, decalcified in 5% formic acid or 10% EDTA, and subsequently dehydrated and embedded in paraffin. Standard frontal sections (7 µm) of the joint tissue were mounted on SuperFrost slides (Menzel-Gläser, Braunschweig, Germany). Hematoxylin and eosin staining was performed to study joint inflammation. The severity of inflammation in the joints was scored on a scale of 0–3 (0 = no cells, 1 = mild cellularity, 2 = moderate cellularity, and 3 = maximal cellularity). To study proteoglycan depletion from the cartilage matrix, sections were stained with safranin O, followed by counterstaining with fast green. Depletion of proteoglycan was determined using an arbitrary scale of 0–3, ranging from normal, fully stained cartilage to destained cartilage that was fully depleted of proteoglycan. Histopathologic changes were scored on three semiserial sections of the joint, with sections spaced 70 µm apart. Scoring was performed in a blind manner by two independent observers. Sections were examined under a light microscope Eclipse E800, (Nikon Instruments Europe, Amstelveen, The Netherlands) and photographed with a Nikon Digital Camera DXM1200 using the software Nikon ACT-1.

### Blood Determinations

Blood samples collected from tails at day 10 were analyzed using an automatic blood analyzer (Scil Vet ABC HORIBA ABX., UK). The following parameters have been determined: white blood cell (WBC) count, differential leukocyte count, red blood cell (RBC) count, hemoglobin concentration, hematocrit, platelet count, mean corpuscular volume, mean corpuscular hemoglobin, and mean corpuscular hemoglobin concentration. Blood samples were centrifuged at 10,000*×g* for 10 min at 4°C to separate the serum. Cytokine content in the mouse serum was analyzed using multiplex Luminex technology. The concentration of IL-1β, IL-6, IL-17, IFN-γ, MCP-1, TNF-α and VEGF was measured according to the manufacturer’s instructions (Millipore, Billerica, MA, USA) with sensitivity of 2.0, 1.8, 0.5, 0.9, 5.3, 1.0 and 0.3 pg/ml, respectively. In addition, the levels of E-selectin, ICAM-1 and PAI-1 with sensitivity of 5.0, 3.0 and 2.0 pg/ml, respectively, have been assessed. Data were analyzed using a Flexmap 3D instrument and Luminex xPONENT® 4.0 Software and Luminex Analyst Program (Luminex Corporation, Austin, Texas, USA). The levels of osteocalcin and RANKL in serum were determined by the LINCOplex™ system (Millipore Iberica, Madrid, Spain), with sensitivity of 7.0 and 3.0 pg/ml, respectively. MMP-3 levels were measured by ELISA (R&D Systems Europe Ltd., Abingdon, UK), with sensitivity of 19.0 pg/ml.

### Measurement of Inflammatory Mediators in Paw Homogenate

Ankles were amputated and homogenized in liquid N_2_ with 1 ml of A buffer pH 7.46 (10 mM HEPES, pH 8, 1 mM EDTA, 1 mM EGTA, 10 mM KCl, 1 mM dithiothreitol, 5 mM NaF, 1 mM Na_3_VO_4_, 1 µg/ml leupeptin, 0.1 µg/ml aprotinine and 0.5 mM phenylmethyl sulfonyl fluoride). The tissue homogenates obtained were sonicated (3×10 sec) and centrifuged at 1200×*g*, 10 min at 4°C. Supernatants were removed and used for determination of mediators. TNF-α and IL-1β were determined by ELISA (R&D Systems Inc., Minneapolis, MN, USA) with a range of detection of 32–2700 and 25–2000 pg/ml, respectively. IL-6, IL-10 and VEGF-A were measured using the ELISA kits from eBioscience Inc. (San Diego, CA, USA), with sensitivity of 4.0, 30.0 and 20.0 pg/ml, respectively. IL-17 and MMP-3 were determined by ELISA kits from R&D Systems Europe Ltd. (Abingdon, UK) with sensitivity of 5.0 and 19.0 pg/ml, respectively. CXCL-1 was determined by ELISA (Promokine, PromoCell GmbH, Heidelberg, Germany, 8–1000 pg/ml). Myeloperoxidase activity was assayed as described previously [37].

### RNA Isolation and Real-time PCR

Fragments of livers isolated from arthritic animals were homogenized in 1 ml of Qiazol (Qiagen GmbH, Hilden, Germany) and mixed with 200 µl of chloroform. The obtained lysates were vortexed, incubated on ice for 15 min, and centrifuged (20 min, 10,000*g* ***,*** 4°C). Then, an upper aqueous phase was collected and subjected to ethanol precipitation. The RNA pellets were dissolved in nuclease-free water. Reverse transcription was performed on 2 µg of total RNA for 1 h at 42°C using oligo(dT) primers (Promega Corporation, Madison, WI, USA) and RevertAid reverse transcriptase (Thermo Scientific, Erembodegem, Belgium). Real-time PCR was carried out using StepOnePlus™ Real-Time PCR Systems (Applied Biosystems, Carlsbad, CA, USA) in a mixture containing SYBR Green PCR Master Mix (Sigma-Aldrich, St. Louis, MO, USA), specific forward and reversed primers (Table S1) and 50 ng of cDNA. EF2 (elongation factor 2) was used as a housekeeping gene. Comparison of gene expression in different samples was performed using ΔC_t_ method.

### Statistical Analysis

Differences between experimental groups were tested using the two-tailed Mann-Whitney U test. *P* values less than 0.05 were considered significant.

## Supporting Information

Table S1 List of primers for gene expression analysis by real-time PCR.
(DOCX)

## References

[1] Choi BM, Pae HO, Jeong YR, Kim YM, Chung HT (2005) Critical role of heme oxygenase-1 in Foxp3-mediated immune suppression. Biochem Biophys Res Commun 327: 1066–1071. 15652505 10.1016/j.bbrc.2004.12.106

[2] Tzima S, Victoratos P, Kranidioti K, Alexiou M, Kollias G (2009) Myeloid heme oxygenase-1 regulates innate immunity and autoimmunity by modulating IFN-beta production. J Exp Med 206: 1167–1179. 19398754 10.1084/jem.20081582 PMC2715044

[3] Kirino Y, Takeno M, Iwasaki M, Ueda A, Ohno S, et al (2005) Increased serum HO-1 in hemophagocytic syndrome and adult-onset Still’s disease: use in the differential diagnosis of hyperferritinemia. Arthritis Res Ther 7: R616–R624. 15899048 10.1186/ar1721 PMC1174958

[4] Rueda B, Oliver J, Robledo G, Lopez-Nevot MA, Balsa A, et al (2007) HO-1 promoter polymorphism associated with rheumatoid arthritis. Arthritis Rheum 56: 3953–3958. 18050210 10.1002/art.23048

[5] Wagener FA, Toonen EJ, Wigman L, Fransen J, Creemers MC, et al (2008) HMOX1 promoter polymorphism modulates the relationship between disease activity and joint damage in rheumatoid arthritis. Arthritis Rheum 58: 3388–3393. 18975324 10.1002/art.23970

[6] Kobayashi H, Takeno M, Saito T, Takeda Y, Kirino Y, et al (2006) Regulatory role of heme oxygenase 1 in inflammation of rheumatoid arthritis. Arthritis Rheum 54: 1132–1142. 16572448 10.1002/art.21754

[7] Kitamura A, Nishida K, Komiyama T, Doi H, Kadota Y, et al (2011) Increased level of heme oxygenase-1 in rheumatoid arthritis synovial fluid. Mod Rheumatol 21: 150–157. 21113640 10.1007/s10165-010-0372-9

[8] Kirino Y, Takeno M, Murakami S, Kobayashi M, Kobayashi H, et al (2007) Tumor necrosis factor alpha acceleration of inflammatory responses by down-regulating heme oxygenase 1 in human peripheral monocytes. Arthritis Rheum 56: 464–475. 17265482 10.1002/art.22370

[9] Devesa I, Ferrandiz ML, Terencio MC, Joosten LA, van den Berg WB, et al (2005) Influence of heme oxygenase 1 modulation on the progression of murine collagen-induced arthritis. Arthritis Rheum 52: 3230–3238. 16200597 10.1002/art.21356

[10] Benallaoua M, Francois M, Batteux F, Thelier N, Shyy JY, et al (2007) Pharmacologic induction of heme oxygenase 1 reduces acute inflammatory arthritis in mice. Arthritis Rheum 56: 2585–2594. 17665394 10.1002/art.22749

[11] Remy S, Blancou P, Tesson L, Tardif V, Brion R, et al (2009) Carbon monoxide inhibits TLR-induced dendritic cell immunogenicity. J Immunol 182: 1877–1884. 19201840 10.4049/jimmunol.0802436

[12] Motterlini R, Otterbein LE (2010) The therapeutic potential of carbon monoxide. Nat Rev Drug Discov 9: 728–743. 20811383 10.1038/nrd3228

[13] Ferrándiz ML, Maicas N, Garcia-Arnandis I, Terencio MC, Motterlini R, et al (2008) Treatment with a CO-releasing molecule (CORM-3) reduces joint inflammation and erosion in murine collagen-induced arthritis. Ann Rheum Dis 67: 1211–1217. 18063671 10.1136/ard.2007.082412

[14] Maicas N, Ferrandiz ML, Devesa I, Motterlini R, Koenders MI, et al (2010) The CO-releasing molecule CORM-3 protects against articular degradation in the K/BxN serum transfer arthritis model. Eur J Pharmacol 634: 184–191. 20184873 10.1016/j.ejphar.2010.02.028

[15] Grundemar L, Ny L (1997) Pitfalls using metalloporphyrins in carbon monoxide research. Trends Pharmacol Sci 18: 193–195. 9226997 10.1016/s0165-6147(97)01065-1

[16] Jozkowicz A, Dulak J (2003) Effects of protoporphyrins on production of nitric oxide and expression of vascular endothelial growth factor in vascular smooth muscle cells and macrophages. Acta Biochim Pol 50: 69–79. 12673348

[17] Kapturczak MH, Wasserfall C, Brusko T, Campbell-Thompson M, Ellis TM, et al (2004) Heme oxygenase-1 modulates early inflammatory responses: evidence from the heme oxygenase-1-deficient mouse. Am J Pathol 165: 1045–1053. 15331427 10.1016/S0002-9440(10)63365-2 PMC1618611

[18] George JF, Braun A, Brusko TM, Joseph R, Bolisetty S, et al (2008) Suppression by CD4+CD25+ regulatory T cells is dependent on expression of heme oxygenase-1 in antigen-presenting cells. Am J Pathol 173: 154–160. 18511516 10.2353/ajpath.2008.070963 PMC2438293

[19] Matsumoto I, Maccioni M, Lee DM, Maurice M, Simmons B, et al (2002) How antibodies to a ubiquitous cytoplasmic enzyme may provoke joint-specific autoimmune disease. Nat Immunol 3: 360–365. 11896391 10.1038/ni772

[20] Wipke BT, Allen PM (2001) Essential role of neutrophils in the initiation and progression of a murine model of rheumatoid arthritis. J Immunol 167: 1601–1608. 11466382 10.4049/jimmunol.167.3.1601

[21] Solomon S, Rajasekaran N, Jeisy-Walder E, Snapper SB, Illges H (2005) A crucial role for macrophages in the pathology of K/B x N serum-induced arthritis. Eur J Immunol 35: 3064–3073. 16180250 10.1002/eji.200526167

[22] Elliott ER, Van Ziffle JA, Scapini P, Sullivan BM, Locksley RM, et al (2011) Deletion of Syk in neutrophils prevents immune complex arthritis. J Immunol 187: 4319–4330. 21918195 10.4049/jimmunol.1100341 PMC3186826

[23] Jacobs JP, Ortiz-Lopez A, Campbell JJ, Gerard CJ, Mathis D, et al (2010) Deficiency of CXCR2, but not other chemokine receptors, attenuates autoantibody-mediated arthritis in a murine model. Arthritis Rheum 62: 1921–1932. 20506316 10.1002/art.27470 PMC2994550

[24] Ji H, Pettit A, Ohmura K, Ortiz-Lopez A, Duchatelle V, et al (2002) Critical roles for interleukin 1 and tumor necrosis factor alpha in antibody-induced arthritis. J Exp Med 196: 77–85. 12093872 10.1084/jem.20020439 PMC2194010

[25] Kaieda S, Tomi C, Oki S, Yamamura T, Miyake S (2007) Activation of invariant natural killer T cells by synthetic glycolipid ligands suppresses autoantibody-induced arthritis. Arthritis Rheum 56: 1836–1845. 17530712 10.1002/art.22714

[26] Pittock ST, Norby SM, Grande JP, Croatt AJ, Bren GD, et al (2005) MCP-1 is up-regulated in unstressed and stressed HO-1 knockout mice: Pathophysiologic correlates. Kidney Int 68: 611–622. 16014038 10.1111/j.1523-1755.2005.00439.x

[27] Matsumoto H, Ishikawa K, Itabe H, Maruyama Y (2006) Carbon monoxide and bilirubin from heme oxygenase-1 suppresses reactive oxygen species generation and plasminogen activator inhibitor-1 induction. Mol Cell Biochem 291: 21–28. 16625420 10.1007/s11010-006-9190-y

[28] Soares MP, Seldon MP, Gregoire IP, Vassilevskaia T, Berberat PO, et al (2004) Heme oxygenase-1 modulates the expression of adhesion molecules associated with endothelial cell activation. J Immunol 172: 3553–3563. 15004156 10.4049/jimmunol.172.6.3553

[29] Vachharajani TJ, Work J, Issekutz AC, Granger DN (2000) Heme oxygenase modulates selectin expression in different regional vascular beds. Am J Physiol Heart Circ Physiol 278: H1613–H1617. 10775141 10.1152/ajpheart.2000.278.5.H1613

[30] Lin HH, Chen YH, Yet SF, Chau LY (2009) After vascular injury, heme oxygenase-1/carbon monoxide enhances re-endothelialization via promoting mobilization of circulating endothelial progenitor cells. J Thromb Haemost 7: 1401–1408. 19426286 10.1111/j.1538-7836.2009.03478.x

[31] Hayes JD, Flanagan JU, Jowsey IR (2005) Glutathione transferases. Annu Rev Pharmacol Toxicol 45: 51–88. 15822171 10.1146/annurev.pharmtox.45.120403.095857

[32] Pham CG, Bubici C, Zazzeroni F, Papa S, Jones J, et al (2004) Ferritin heavy chain upregulation by NF-kappaB inhibits TNFalpha-induced apoptosis by suppressing reactive oxygen species. Cell 119: 529–542. 15537542 10.1016/j.cell.2004.10.017

[33] Balla G, Jacob HS, Balla J, Rosenberg M, Nath K, et al (1992) Ferritin: a cytoprotective antioxidant strategem of endothelium. J Biol Chem 267: 18148–18153. 1517245

[34] Shah D, Wanchu A, Bhatnagar A (2011) Interaction between oxidative stress and chemokines: possible pathogenic role in systemic lupus erythematosus and rheumatoid arthritis. Immunobiology 216: 1010–1017. 21601309 10.1016/j.imbio.2011.04.001

[35] Was H, Sokolowska M, Sierpniowska A, Dominik P, Skrzypek K, et al (2011) Effects of heme oxygenase-1 on induction and development of chemically induced squamous cell carcinoma in mice. Free Radic Biol Med 51: 1717–1726. 21867749 10.1016/j.freeradbiomed.2011.07.025 PMC3192260

[36] Poss KD, Tonegawa S (1997) Heme oxygenase 1 is required for mammalian iron reutilization. Proc Natl Acad Sci USA 94: 10919–10924. 9380735 10.1073/pnas.94.20.10919 PMC23531

[37] Payá M, Terencio MC, Ferrándiz ML, Alcaraz MJ (1996) Involvement of secretory phospholipase A_2_ activity in the zymosan rat air pouch model of inflammation. Br J Pharmacol 117: 1773–1779. 8732290 10.1111/j.1476-5381.1996.tb15353.x PMC1909548

