# Supplementary material for: Heme Oxygenase-1 Regulates the Progression of K/BxN Serum Transfer Arthritis
Source: PLoS One. 2012 Dec 20;7(12):e52435. doi: 10.1371/journal.pone.0052435 (PMC3527514; doi:10.1371/journal.pone.0052435)
Supplement: Table S1 — List of primers for gene expression analysis by real-time PCR. (DOCX) [file pone.0052435.s001.docx]

**Table S1.**

| **gene** | **forward** | **reversed** |
| --- | --- | --- |
| **EF2** | 5’-GCGGTCAGCACAATGGCATA | 5’-GACATCACCAAGGGTGTGCAG |
| **Catalase** | 5′-TGACATGGTCTGGGACTTCTGG | 5′-TTGATGCCCTGGTCGGTCTT |
| **GSTA-1** | 5′-CCCCTTTCCCTCTGCTGAAG | 5′-TGCAGCTTCACTGAATCTTGAAAGC |
| **GR** | 5′-GGCACTTGCGTGAATGTTGG | 5′-GGCATCCCTTTTCTGCTTGATG |
| **Ferritin** | 5′-CTTCGAGCCTGAGCCCTTTG | 5′-CAGGTTGATCTGGCGGTTGA |
| **Thrx-2** | 5′-GCTAGAGAAGATGGTCGCCAAGCAG | 5′-TCCTCGTCCTTGATCCCCACAAACTTG |
| **SOD-2** | 5′-AATCTCAACGCCACCGAGGA | 5′-TCTCCTTTGGGTTCTCCACCA |
